# High use of private providers for first healthcare seeking by drug-resistant tuberculosis patients: a cross-sectional study in Yangon, Myanmar

**DOI:** 10.1186/s12913-018-3077-y

**Published:** 2018-04-11

**Authors:** Sucitro Dwijayana Sidharta, Jason Dean-Chen Yin, Joanne Su-Yin Yoong, Mishal Sameer Khan

**Affiliations:** 1 Saw Swee Hock School of Public Health, National University of Singapore, 12 Science Drive 2, #10-01, Singapore, 117549 Singapore; 2 Center for Economic and Social Research, University of Southern California, 1909 K St NW, Suite 530, Washington, DC, 20006-1101 USA; 3 London School of Hygiene and Tropical Medicine, 15–17 Tavistock Place, London, WC1H 9SH UK

**Keywords:** Tuberculosis, Myanmar, Drug-resistance, Health systems, Private health sector

## Abstract

**Background:**

Drug resistance is a growing challenge to tuberculosis (TB) control worldwide, but particularly salient to countries such as Myanmar, where the health system is fragmented across the public and private sector. A recent systematic review has identified a critical lack of evidence for local policymaking, particularly in relation to drivers of drug-resistance that could be the target of preventative efforts. To address this gap from a health systems perspective, our study investigates the healthcare-seeking behavior and preferences of recently diagnosed patients with drug-resistant tuberculosis (DR-TB), focusing on the use of private versus public healthcare providers.

**Methods:**

The study was conducted in ten townships across Yangon with high DR-TB burden. Patients newly-diagnosed with DR-TB by GeneXpert were enrolled, and data on healthcare-seeking behavior and socio-economic characteristics were collected from patient records and interviews. A descriptive analysis of healthcare-seeking behavior was followed by the investigation of relationships between socio-economic factors and type of provider visited upon first feeling unwell, through univariate logistic regressions.

**Results:**

Of 202 participants, only 8% reported first seeking care at public facilities, while 88% reported seeking care at private facilities upon first feeling unwell. Participants aged 25–34 (Odds Ratio = 0.33 [0.12–0.95]) and males (Odds Ratio = 0.39 [0.20–0.75]) were less likely to visit a private clinic or hospital than those aged 18–24 and females, respectively. In contrast, participants with higher income were more likely to utilize private providers. Prior to DR-TB diagnosis, 86% of participants took medications from private providers. After DR-TB diagnosis, only 7% of participants continued to take medications from private providers.

**Conclusion:**

In urban Myanmar, most patients shifted to being managed exclusively in the public sector after being formally diagnosed with DR-TB. However, since the vast majority of DR-TB patients first visited private providers in the period leading to diagnosis, related issues such as unregulated quality of care, potential delays to diagnosis, and lack of care continuity may greatly influence the emergence of drug-resistance. A greater understanding of the health system and these healthcare-seeking behaviors may simultaneously strengthen TB control programmes and reduce government and out-of-pocket expenditures on the management of DR-TB.

## Background

Although tuberculosis (TB) mortality has fallen by 47% between 1990 and 2015, TB remains one of the world’s deadliest infectious diseases. In 2016 there were still approximately 1.3 million deaths due to TB (among HIV-negative), surpassing an estimated 1.0 million due to HIV / AIDS. Moreover, in recent years, public health experts have become increasingly concerned about the emergence and spread of drug-resistant strains of TB, particularly those that are not controlled by one or both most effective anti-TB drugs, rifampicin and isoniazid (referred to as DR-TB going forward). Growing drug-resistance is a major hurdle to global TB control, threatening to stall or even reverse the declines in TB incidence and mortality [1].

Recent WHO figures indicate that 19% of previously-treated TB cases and 4.1% of new TB cases globally were DR-TB. This burden is unequally distributed, both spatially and economically: of the 600,000 DR-TB cases emerging in 2016, 91% originated in just 30 countries, 22 of which are classified as low or lower-middle income [2]. In most of these countries, government spending on health per capita is relatively low, and out-of-pocket expenditures exceed 15% of total healthcare costs (well over the suggested benchmark threshold for universal health coverage) [1, 3]. These and other systemic barriers - such as high costs of formal diagnosis and treatment; limited accessibility; poor management and infrastructure; and weak regulatory oversight - result in health systems that are already overstrained yet faced with a growing and increasingly complex burden of diseases, creating ideal conditions for the continued rise of drug-resistance [4]. In addition to these macro-level factors, however, the role of patients and their behavior is also critical. Individual beliefs and preferences, as well as household social and financial constraints, can lead to suboptimal risk-taking, healthcare-seeking and compliance-related behaviors, the consequences of which may be further exacerbated by weaknesses in the underlying health system; for instance, poor health literacy coupled with limited access and high prices can lead to delays in diagnosis and treatment [5–7]. Alternatively, lack of patient motivation to comply with treatment, combined with low levels of monitoring will lead to treatment failure and contribute to the emergence of DR-TB [8, 9].

Myanmar exemplifies the case of a lower-income country with a high DR-TB burden. The TB incidence rate is 361 per 100,000 population, approximately 2.5 times higher than the global average. DR-TB incidence is approximately 13,000 per year, placing Myanmar among the highest DR-TB burden countries in the world. Overall, 27% of previously treated TB cases and 5.1% of new TB cases were estimated to be DR-TB, higher than both the global and regional average [1]. Despite progress towards eliminating infectious diseases per the “Good Health and Wellbeing” portion of the Sustainable Development Goals, Myanmar’s health system is still developing. Although international attention and funding has turned towards Myanmar in recent years, low levels of human capital and government spending in healthcare (3.4% of general government spending in 2014–2015) [10] continue to constrain access, coverage and service quality. A recent systematic review in Myanmar identified a critical lack of evidence on TB in the local context to inform policy decisions. Specifically, the study highlighted a need for funders and policymakers to have better information about drivers of drug-resistance that could be the target of preventative efforts and health systems strengthening [11]. To address this gap, our present study investigates the healthcare-seeking behavior and preferences of recently diagnosed DR-TB patients.

## Methods

### Study setting

Myanmar is a lower-middle income country with a population of 53 million as of 2016. It is bordered by China, India, Bangladesh, Thailand, and Laos. Healthcare services in Myanmar are provided by both the private and public sector. The public health sector is overseen by the Ministry of Health and Sports, which manages public services such as those provided by the National TB Programme (NTP). TB control activities are implemented through Yangon’s Township Health Departments (THDs), which are the main health facilities providing primary healthcare, including TB diagnosis, case registration, and treatment provision. Private sector providers include hospitals, pharmacies, small clinics, and informal drug sellers and dispensaries, some of which are not formally registered or regularly monitored. Many private healthcare providers or organizations that work in partnership with the government– such as the Myanmar Medical Association (MMA) and Population Service International (PSI) – are regulated and work closely with the NTP to provide standardized treatment [12]. This cross-sectional study was conducted in ten THDs across Yangon, identified by the NTP as having a high DR-TB burden.

### Participants

Our study enrolled all newly-diagnosed adult pulmonary DR-TB patients with confirmed rifampicin resistance based on GeneXpert results at the THDs. Recruitment was conducted over a six-month period from September 2014 to March 2015. The exclusion criteria were: age < 18 years; known pregnancy at time of data collection; residence outside Yangon or in Yangon < 3 months; and extra-pulmonary TB only.

### Data collection

After verifying eligibility and willingness to participate (through written consent), we retrospectively collected data from DR-TB patients 3 months after diagnosis at THDs. All DR-TB patients had previously been treated for TB. Data were collected by administering a pre-piloted, structured questionnaire at the participant’s home, collecting data about participant’s socio-economic status, symptoms, treatment adherence, healthcare-seeking behavior, and treatment support. Questions about healthcare-seeking behavior focused on decisions made when patients had a typical mild or moderate illness (such as a cough) and not specifically for their recent TB symptoms; this allowed data collection on healthcare-seeking before patients may have been aware that they had TB. A local data collector fluent in the native language was trained to administer the questionnaires and assigned to each township.

### Data management

Data collected on paper records was manually checked at the local study site for accuracy by the study supervisor. Data were then entered into Epidata (Version 3.1, The EpiData Association, Odense, Denmark) and checked for quality through blinded double entry and restricting out-of-range values. The raw data was then transferred to Stata (version 10, Stat Corporation, College Station, TX) for management and statistical analysis.

### Statistical analysis

We conducted a descriptive analysis, reporting numbers, percentages, and 95% confidence intervals (of the proportion) for categorical variables summarizing individual and household socio-economic characteristics, as well as key healthcare seeking behaviors. We then investigated the relationship between patients’ socio-economic factors and healthcare seeking behavior in terms of visits to a private provider when initially feeling unwell. Specifically, the outcome variable was whether study participants self-reported that they usually visit a private clinic or hospital upon first feeling unwell (with any ailment). Univariate logistic regressions were conducted, and results were stated in terms of odds ratios and 95% confidence intervals.

## Results

Over the study period, 205 eligible participants with DR-TB were diagnosed at the ten THDs. Three refused to participate, resulting in 202 DR-TB participants being included in the final analysis. Demographic and socio-economic characteristics of the study participants are summarized in Table 1. Approximately 70% of the participants are aged between 25 and 54, representing the most active socio-economic group, and the number of males was roughly double that of females. Although 12 participants died before the interview, they remained in our analysis to avoid bias from removing this subset, and information about healthcare-seeking behavior was collected from their family members.

**Table 1:** Demographic and socio-economic characteristics of DR-TB participants (*N* = 202)

Characteristics	N (%)	95% CI
Age		
18–24	26 (13%)	(9–18%)
25–34	57 (28%)	(22–35%)
35–44	43 (21%)	(16–28%)
45–54	38 (19%)	(14–25%)
55–64	22 (11%)	(7–16%)
65>	16 (8%)	(5–13%)
Female	64 (32%)	(26–39%)
Ethnic group		
Bamar	169 (84%)	(78–88%)
Mixed	6 (3%)	(1–7%)
Ethnic minority	27 (13%)	(9–19%)
Religion		
Buddhist	185 (92%)	(87–95%)
Christian	10 (5%)	(3–9%)
Muslim	7 (4%)	(2–7%)
Marital status		
Single never married	61 (30%)	(24–37%)
Married / cohabitating	121 (60%)	(53–67%)
Divorced / separated / widowed	20 (10%)	(7–15%)
Occupation		
Dependent on earnings of a family member	44 (22%)	(17–28%)
Daily wage earner	5 (3%)	(1–6%)
Self-employed or private employee	61 (30%)	(24–37%)
Government employee	13 (6%)	(4–11%)
Unemployed	71 (35%)	(29–42%)
Retired or other	8 (4%)	(2–8%)
Education level		
None or less than primary	35 (17%)	(13–23%)
Primary school completed	54 (27%)	(21–33%)
Middle school completed	59 (29%)	(23–36%)
High school completed or graduated	54 (27%)	(21–33%)
Household income in kyat (and USD)^a^		
0/100000 (0/91)	38 (19%)	(14–25%)
100,001/150000 (91/136)	38 (19%)	(14–25%)
150,001/200000 (136/18)	46 (23%)	(18–30%)
200,001/300000 (181/271)	40 (20%)	(15–26%)
300,001/max (271/max)	33 (16%)	(12–22%)
Missing	7 (4%)	(2–7%)
Type of dwelling		
Permanent	119 (59%)	(52–66%)
Informal	83 (41%)	(35–48%)
Patient still alive		
Yes	190 (94%)	(90–97%)
No	12 (6%)	(3–10%)

^a^1 USD = 1105 Kyat on 15 June 2015 (Source: Central Bank of Myanmar [30])

As expected, most participants are of the Bamar ethnic group (84%), the dominant ethnic group in Myanmar, and are Buddhist (92%), the dominant religion in the country. Of the 202 participants, 41% reported living in informal housing (without official address and usually built of impermanent materials rather than permanent structures).

Table 2 shows the healthcare-seeking behavior of DR-TB participants in our study. Only 8% of study participants reported a preference to seek care at public facilities upon first feeling unwell (with any ailment). Most participants utilized the private sector, with 62% visiting private clinics and hospitals, and 26% visiting private pharmacies or drug sellers. A small number (3%) went to informal drug sellers or traditional healers. For healthcare-seeking behavior in the past 2 years, 72% reported having seen a private doctor, while use of traditional healers was low (7%). Before diagnosis of DR-TB was confirmed at a THD, most participants used medications from a private doctor or pharmacy (86%). Following the diagnosis of DR-TB – at approved government facilities (i.e. NTP or the public sector) – only 34 (17%) participants still visited private doctors and only 8 out of 34 participants (24%) took treatment from them. When considering the full range of private providers (not only private doctors, but including pharmacies and traditional healers as well), 15 (7%) participants reported taking any treatment from these providers following diagnosis.

**Table 2:** Healthcare-seeking behavior of DR-TB participants (N = 202)

Healthcare-seeking behavior	N (%)	95% Confidence Interval (of the proportion)
Type of health care provider visited when first feeling unwell		
Private clinic/hospital	126 (62%)	(55–69%)
Public clinic/hospital	16 (8%)	(5–13%)
Pharmacy (formal)	53 (26%)	(21–33%)
Informal drug stores	3 (2%)	(1–5%)
Informal non-medical provider/traditional healer	1 (1%)	(1–4%)
Nobody	3 (2%)	(1–5%)
Visited private doctor in the past 2 years?		
No	56 (28%)	(22–34%)
Yes	146 (72%)	(66–78%)
Visited traditional healer in the past 2 years?		
No	188 (93%)	(89–96%)
Yes	14 (7%)	(4–11%)
Taken any medication from private/pharma before DR-TB diagnosis?		
No	29 (14%)	(10–20%)
Yes	173 (86%)	(80–90%)
Visited private doctor for DR-TB concerns since diagnosis?		
No	168 (83%)	(77–88%)
Yes	34 (17%)	(12–23%)
Taken *any* treatment for DR-TB from private doctor or pharmacy *since* diagnosis?		
No	194 (96%)	(92–98%)
Yes	8 (4%)	(2–8%)
Taken *any* treatment for DR-TB from traditional healer *since* diagnosis?		
No	194 (96%)	(92–98%)
Yes	8 (4%)	(2–8%)
Taken *any* treatment for DR-TB in private sector (private doctor, pharmacy, traditional healer) *since* diagnosis?		
No	187 (93%)	(88–95%)
Yes	15 (7%)	(5–12%)

Table 3 shows the results of univariate logistic regression with the outcome being whether participants reported first seeking care at a private clinic or hospital when they are unwell. Participants aged 25–34 (OR: 0.33 [0.12–0.95]) were significantly less likely to go to a private clinic or hospital than those aged between 18 and 24. Male participants (OR: 0.39 [0.20–0.75]) were also less likely to go to a private clinic or hospital compared to female participants. Participants with greater income had higher odds of visiting private providers, in particular those with incomes above 300,000 Kyat (OR: 5.040 [1.60–15.84]).

**Table 3:** Logistic regression on the type of healthcare provider visited when first time feeling unwell (private clinic/hospital: yes, others: no, N = 202)

Characteristics	Yes	No	Odds ratio	95% Confidence interval
Age				
18–24	20	6	1	1
25–34	30	27	0.33*	(0.12,0.95)
35–44	28	15	0.56	(0.19,1.69)
45–54	25	13	0.58	(0.19,1.79)
55–64	11	11	0.30	(0.09,1.03)
65>	12	4	0.90	(0.21,3.85)
Sex				
Woman	49	15	1	1
Man	77	61	0.39**	(0.20,0.75)
Ethnic group				
Bamar	106	63	1	1
Mixed	4	2	1.19	(0.21,6.68)
Ethnic minority	16	11	0.86	(0.38,1.98)
Religion				
Buddhist	115	70	1	1
Christian	7	3	1.42	(0.36,5.67)
Muslim	4	3	0.81	(0.18,3.73)
Marital status				
Single never married	42	19	1	1
Married/cohabitating	73	48	0.69	(0.36,1.32)
Divorced/separated/widowed	11	9	0.55	(0.20,1.56)
Occupation				
Dependent on earnings of a family member	36	8	1	1
Daily wage earner	4	1	0.89	(0.09,9.06)
Self-employed or private employee	35	26	0.30*	(0.12,0.75)
Government employee	7	6	0.26*	(0.07,0.98)
Unemployed	40	31	0.29**	(0.12,0.70)
Retired or other	4	4	0.22	(0.05,1.08)
Education level				
None or Less than primary	20	15	1	1
Primary school completed	35	19	1.38	(0.58,3.30)
Middle school completed	34	25	1.02	(0.44,2.38)
High school completed or graduated	37	17	1.63	(0.68,3.94)
Household incomed in kyat (and USD)^a^				
0/100000 (0/91)	20	18	1	1
100,001/150000 (91/136)	20	18	1	(0.41,2.46)
150,001/200000 (136/181)	27	19	1.28	(0.54,3.04)
200,001/300000 (181/271)	25	15	1.50	(0.61,3.70)
300,001/max (271/max)	28	5	5.04**	(1.60,15.84)
Missing	6	1	5.40	(0.59,49.26)
Permanent/informal dwelling				
Permanent	79	40	1	1
Informal	47	36	0.66	(0.37,1.18)

^a^1 USD = 1105 Kyat on 15 June 2015 (Source: Central Bank of Myanmar [30])

*: *p* < 0.05, **: *p* < 0.01

## Discussion

Drug-resistant TB is a challenge for resource-constrained settings where healthcare is provided by a range of healthcare providers in the public and private sectors. In countries such as Myanmar, in addition to low government funding [10], fragmented healthcare systems may contribute to the emergence of drug-resistance and reduce capacity to manage and diagnose drug-resistant cases [13–15]. To our knowledge, ours is the first study to quantitatively evaluate the health-seeking behavior of DR-TB patients in Myanmar, focusing on behavior with respect to the use of private healthcare providers.

We found that the vast majority of participants prefer to utilize the private sector and take prescribed medications from private providers when they start feeling unwell. Similar findings are reported in other Southeast and South Asian countries with dominant private sectors [16, 17]. It is important to highlight the heterogeneity of the provider landscape for allopathic health services in Myanmar and other regional countries; the private healthcare sector includes independently operating, usually unregulated GPs, pharmacies, and hospitals as well as international and local non-government organizations (NGOs) such as Population Services International (PSI). Private GPs also undertake dual practice which means that they are employed in public sector and see private patients outside of office hours as well [18]. Thus, the diverse entities that constitute the private sector add to its complexity. In contrast to the high use of allopathic private providers, we found limited use of traditional or complementary medicine practitioners in Yangon. Finally, regardless of which provider in the private sector participants initially choose, after a diagnosis of DR-TB in the public health sector, we found that the majority switch to using the public sector and do not continue to be treated by private healthcare providers.

A highly-fragmented pathway to care with a large de-facto role for the private sector, as found in our study, can facilitate the development of DR-TB. The quality of care received at unregulated private providers is poorly studied or documented through the routine monitoring systems that are applied in the public sector. There is some evidence indicating that diagnosis and treatment in the private sector can deviate from guidelines for appropriate patient management [19] and that poor treatment compliance in the private sector contributes to drug-resistant TB [20–22]. We do not know the length of delay to diagnosis of DR-TB in Myanmar; a long delay to initiating treatment may result in spread of infection and this should be monitored among newly diagnosed DR-TB patients.

From the perspective of patient choice, use of the private or public sector is determined by both push and pull factors. Few studies in Myanmar have looked at the motivations for initially using private as opposed to public healthcare. One study found most people went to a private GP nearby since it was more convenient and was encouraged by former patients who had been cured of the disease. Long waiting times and perceived prolonged risk to exposure in the waiting rooms were also reported as deterrents to the use of the public sector [18].

In our study, we found that women were more likely to use private providers, consistent with other research that also found that male TB patients outnumbered females in Yangon’s public health sector [23]. Evidence from other settings indicates that women may experience greater stigma from TB or challenges in travelling long distances to reach healthcare, and private providers may be perceived to allow greater patient privacy and be more conveniently located [24–26]. Further research is needed to explain other demographic differences, such as age-related disparities in seeking care from private providers.

The most likely main determinant of switching to the public sector after diagnosis with DR-TB is the high cost of future visits in the private sector, because DR-TB requires expensive medication [27, 28]. A study in Cambodia also found that patients would initially visit private healthcare providers and switch to the public sector once they knew they had TB, because they wanted to access free TB treatment which would not be available in the private sector [29]. Proximity and access to health public facilities did not appear to be a major barrier in our study in an urban setting, with 78% of the participants able to reach NTPs within 30 min.

A major strength of this study is the collection of data via detailed, face-to-face interviews with all newly-diagnosed DR-TB participants in the studied townships, which has not previously been collected in Myanmar. However, our study is limited in that we only focus on selected townships within Yangon, and so our findings could more representative of urban settings. For example, the finding that few DR-TB patients used traditional healers may differ in rural settings. Costs of seeking care are also missing from our dataset as we did not receive ethical approval to collect this information. Likewise, our study lacks data on delays to diagnosis of DR-TB, as well as the time period between first seeking care in the private healthcare sector and shifting to the public sector. Lastly, there is considerable diversity in private sector providers that we have not investigated. Differences in quality of care, referral practices and costs may differ between registered/regulated private provider and informal or untrained providers.

## Conclusions

As funding increases for infectious disease control in Myanmar, this research provides initial evidence for policy making and resource allocation decisions [10, 11]. Specifically, our study indicates that private allopathic healthcare providers are the first point of contact when patients are unwell, and they can therefore play a major positive or negative role in healthcare provision in urban settings. Investments that can improve the quality of care, delays to diagnosis, and continuity of care within the private sector, or coordination between the public and private sector, have the potential to impact a large proportion of patients and reduce the growth in drug-resistance in Myanmar. The current study adds to the limited evidence on healthcare-seeking behavior among DR-TB patients in Asian settings with pluralistic health systems. Future studies should examine what motivates patients to use the private sector as the first point on contact when ill, and subsequently understand the quality of care for different types of providers within this sector. Longitudinal studies can help elucidate factors associated with timing of diagnosis and treatment uptake, and treatment adherence, which will support the design and funding of effective interventions in the future.

## Abbreviations

AIDS / HIV: Acquired Immunodeficiency Syndrome / Human Immunodeficiency Virus
CI: Confidence interval
DR-TB: Drug-resistant tuberculosis
GP: General practitioner
MMA: Myanmar Medical Association
NGO: Non-governmental Organization
NTP: National Tuberculosis Programme
OOP: Out of pocket
OR: Odds ratio
PSI: Population Service International
TB: Tuberculosis
THD: Township Health Department
USD: United States Dollars
WHO: World Health Organization
